# Melatonin Can Modulate the Effect of Navitoclax (ABT-737) in HL-60 Cells

**DOI:** 10.3390/antiox9111143

**Published:** 2020-11-18

**Authors:** Alexey Lomovsky, Yulia Baburina, Irina Odinokova, Margarita Kobyakova, Yana Evstratova, Linda Sotnikova, Roman Krestinin, Olga Krestinina

**Affiliations:** Institute of Theoretical and Experimental Biophysics, Russian Academy of Sciences, 142290 Pushchino, Russia; lomovskyalex@gmail.com (A.L.); byul@rambler.ru (Y.B.); odinokova@rambler.ru (I.O.); ritaaaa@gmail.com (M.K.); yannaevstratova@gmail.com (Y.E.); linda_sotnikova@mail.ru (L.S.); rkrestinin@bk.ru (R.K.)

**Keywords:** acute promyelocytic leukemia, HL-60 cells, melatonin, navitoclax (ABT-737), permeability transition pore, apoptosis, endoplasmic reticulum stress, autophagy

## Abstract

Melatonin (*N*-acetyl-5-methoxytryptamine MEL) is an indolamine that has antioxidant, anti-inflammatory and anti-tumor properties. Moreover, MEL is capable of exhibiting both anti-apoptotic and pro-apoptotic effects. In the normal cells, MEL possesses antioxidant property and has an anti-apoptotic effect, while in the cancer cells it has pro-apoptotic action. We investigated the combined effect of MEL and navitoclax (ABT-737), which promotes cell death, on the activation of proliferation in acute promyelocytic leukemia on a cell model HL-60. The combined effect of these compounds leads to a reduction of the index of mitotic activity. The alterations in the level of anti- and pro-apoptotic proteins such as BclxL, Bclw, Mcl-1, and BAX, membrane potential, Ca^2+^ retention capacity, and ROS production under the combined action of MEL and ABT-737 were performed. We obtained that MEL in combination with ABT-737 decreased Ca^2+^ capacity, dropped membrane potential, increased ROS production, suppressed the expression of anti-apoptotic proteins such as BclxL, Bclw, and Mcl-1, and enhanced the expression of pro-apoptotic BAX. Since, MEL modulates autophagy and endoplasmic reticulum (ER) stress in cancer cells, the combined effect of MEL and ABT-737 on the expression of ER stress and autophagy markers was checked. The combined effect of MEL and ABT-737 (0.2 μM) increased the expression of protein kinase R (PKR)-like endoplasmic reticulum kinase (PERK), leading to a decrease in the level of binding immunoglobulin protein (BIP) followed by an increase in the level of C/EBP homologous protein (CHOP). In this condition, the expression of ERO1 decreased, which could lead to a decrease in the level of protein disulfide isomerase (PDI). The obtained data suggested that melatonin has potential usefulness in the treatment of cancer, where it is able to modulate ER stress, autophagy and apoptosis.

## 1. Introduction

Melatonin (*N*-acetyl-5-methoxytryptamine, MEL) is an indolamine that is produced in humans and animals during the dark. The main producer of this hormone is the pineal gland but it is also produced in some organs such as the skin, bone marrow, retina, and gastrointestinal tract [1,2]. MEL has anti-inflammatory, antioxidant, and oncostatic effects [3,4]. It is known that MEL is capable of exhibiting both anti-apoptotic and pro-apoptotic properties. The reason for this effect of MEL lies in the mechanisms of its action [5,6]. Several studies have shown that MEL has important oncostatic properties due to receptor-dependent and receptor-independent mechanisms [7]. There are two membrane receptors that were pharmacologically characterized, later cloned, and are now referred to as MT1 and MT2 receptors. Both members of the transmembrane superfamily are associated with G-protein receptors [6,8,9]. The receptor-dependent action of MEL leads to an anti-proliferative effect, whereas the receptor-independent action of MEL is associated with antioxidant activity, regulation of apoptosis, tumor metabolism and cancer immunity, inhibition of angiogenesis and migration, and prevention of circadian disorders [7,10]. As a pro-apoptotic agent, MEL affects the permeabilization of the mitochondrial inner membrane, which leads to the opening of the mitochondrial permeability transition pore (mPTP) [5]. mPTP was shown to be a regulator of cell death [11]. Oxidative stress and Ca^2+^ threshold concentration are considered as inducers of mPTP [12]. Loss of mitochondrial inner membrane permeability leads to a rapid drop in the mitochondrial membrane potential (ΔΨm), depolarization of the membrane, rupture of the outer mitochondrial membrane, and ultimately to cell death [13]. Endoplasmic reticulum stress (ER stress) may trigger the opening of mPTP [14].

Generally accepted that the endoplasmic reticulum (ER) is one of the most important organelles in the cell because it is able to regulate Ca^2+^ accumulation, lipid synthesis and protein folding [15,16]. Yina Chen and coauthors showed that MEL reduces the inflammatory response; the expression of proteins associated with ER stress, and inhibits cell apoptosis. Researchers concluded that MEL attenuated the inflammatory response by inhibiting the activation of ER stress and suppressing RAW264.7 macrophage apoptosis [17]. Moreira and others observed that MEL also elicited an ER stress response in cytosolic extracts obtained by homogenizing liver tissue from hepatocarcinogenic rats with MEL administration [18]. The authors concluded that the observed effects might be related to apoptosis. MEL (1 mM) suppressed cell viability through the overproduction of superoxide and increased the expression of cellular prion protein in colorectal cancer cells. The inhibition of cellular prion protein-enhanced MEL-mediated superoxide accumulation and activated reactive oxygen species (ROS)-mediated ER stress [19]. ER stress can result in the induction of autophagy, which is a dynamic process triggered by the self-digestion of damaged organelles and misfolded proteins in cells [20].

It is generally known that proteins of the Bcl-2 family prevent apoptosis by protecting the integrity of the outer mitochondrial membrane by binding to BAX/Bak and disrupting the interaction between proteins of the Bcl-2 family upstream of the caspases [21,22]. Recently, an inhibitor of Bcl_XL_ that also inhibits Bcl-2 and Bcl_w_, called navitoclax (ABT-737), has been described. In these proteins, ABT-737 binds to the hydrophobic groove and prevents the sequestration of pro-apoptotic proteins such as BAD and BIM [23]. It was found that ABT-737 promotes cell death after treatment with agents that induce pro-apoptotic signals [23]. ABT-737 also exhibits significant monotherapeutic activity against myeloma, leukemia, and small cell lung cancer cells in vitro [23,24].

Earlier, we showed that MEL in combination with a reduced concentration of retinoic acid or cytarabine increased the cytotoxicity of retinoic acid toward HL-60 cells and suppressed the expression of anti-apoptotic Bcl-2 protein [25,26]. MEL in combination with retinoic acid or cytarabine decreased the expression of the subunits of respiratory complexes, thereby reducing their activity. Our findings suggested that MEL is able to enhance the effects of other chemotherapeutic agents and can be used in new cancer therapy strategies. In the present work, the combined effect of MEL (at a pharmacological concentration—1 mM) and ABT-737 (0.2 µM) on the activation of proliferation in HL-60 cells was investigated, and an analysis of the cell cycle in these cells was performed. In addition, we analyzed the alterations in the level of anti- and pro-apoptotic proteins such as Bcl_xL_, Bcl_w_, Mcl-, and BAX, respectively. The effect of MEL in combination with ABT-737 (0.2 µM) on the membrane potential and ROS production was estimated. Since there is a relationship between apoptosis, autophagy and ER stress [27], we investigated the change in markers for ER stress and autophagy in our experimental conditions.

## 2. Materials and Methods

### 2.1. Chemicals and Reagents

Melatonin (*N*-acetyl-5-methoxytryptamine, MEL), resazurin sodium salt, propidium iodide (PI), and a protease/phosphatase inhibitor cocktail were from Sigma-Aldrich (St. Louis, MO, USA). Navitoclax (ABT-737) was Selleckchem (Houston, TX, USA).

### 2.2. Cell Culture and Treatments

Human promyelocytic leukemia HL-60 cells (CCL-240) were from ATCC (Manassas, VA, USA). The cells were cultured in RPMI 1640 medium (Sigma-Aldrich, USA) to which 20% fetal bovine serum (Gibco, Grand Island, NY, USA) and 40 μg/mL gentamicin sulfate (Sigma-Aldrich, USA) was added at 37 °C at 95% humidity and 5% CO_2_. HL-60 cells were treated with different concentrations of MEL (from 20 nM to 1 mM) and ABT-737 (from 3 × 10^−3^ to 20 mM) for 24 h.

### 2.3. Cell Viability Assay

Cell viability was assessed using a cell viability assay with resazurin. Cells were seeded in a 96-well plate at 5 × 10^3^ cells per well. After 24 h, MEL and ABT737 at specific doses were added to HL-60 cells. Twenty-four hours after treatment, resazurin (Sigma-Aldrich) at a final concentration of 100 μg/mL was added to each well in the cells, and incubated for 4 h at 37 °C. The fluorescence intensity was measured using an Infinite F200 microplate reader (Tecan, Grodig, Austria) at an excitation wavelength of 535 nm and an emission wavelength of 595 nm. Data are presented as a percentage of control cells (untreated samples).

### 2.4. Determination of the Mitotic Index

To determine mitotic activity, the cells were incubated for 96 h under various conditions, centrifuged (250× *g*, 10 min), resuspended in PBS, and fixed with 70% ethanol (30 min, CT). The fixed cells were stained with bis-benzimide H33342 (Sigma-Aldrich, Saint Louis, MO, USA) and the cell mitotic index was calculated using a DM 6000 fluorescence microscope (Leica, Wetzlar, Germany). The mitotic index (MI) was determined by the formula MI = (P + M + A + T)/N, where P + M + A + T is the sum of all cells in the phases: prophase, metaphase, anaphase and telophase, respectively, and N is the total number of cells.

### 2.5. Cell Growth Assays

Cell growth was assessed by counting cells at various time points after treatment. Cells were centrifuged for 10 min at 250× *g* and washed with PBS, and stained with 0.4% trypan blue (Sigma-Aldrich, Saint Louis, MO, USA) to assess cell numbers and cell viability. In each experiment, three samples were counted per group, and the experiments were performed in at least three replicates.

### 2.6. Measurement of Intracellular ROS Generation

Intracellular oxidative activity was assessed by the fluorescence of the 2′-7′-dichlorodihydrofluorescein diacetate (DCFH-DA) probe (Ex-485nm/Em-530nm). To assess the intracellular ROS generation, 10 μM DCFH-DA was added to the medium with cells (4 × 10^5^ cells/mL) and incubated in the dark in a CO_2_ incubator for 10 min. After staining, cells were washed with PBS once. H_2_O_2_ (1 mM) was used as a positive control. Determination of oxidative activity was performed using a BD Accuri C6 flow cytometer.

### 2.7. The Mitochondrial Membrane Potential

The mitochondrial membrane potential was assessed using the fluorescent dye 3,3′-dihexyloxacarbocyanine iodide (DiOC6(3)) (Ex-482 nm/Em-501 nm). To assess the change in the mitochondrial membrane potential of cells, 10 nM DiOC6(3) was added to a cell suspension in a culture medium (10^6^ cells/mL) and incubated in the dark in a CO_2_ incubator for 30 min. After staining, cells were washed with PBS once. Saponin (0.5%) was used as a positive control. Determination of changes in the mitochondrial membrane potential was performed using a BD Accuri C6 flow cytometer.

### 2.8. Ca^2+^-Retention Capacity of Mitochondria in Permeabilized Cells

Ca^2+^ fluxes were recorded in a multifunctional chamber with a built-in Ca^2+^ electrode, a computerized recording system Record 4 (Institute of Theoretical and Experimental Biophysics, Russian Academy of Sciences, Pushchino, Russia). Cells (4 × 10^6^/mL) were treated with 0.007% digitonin to increase the permeability of the plasma membrane. Ca^2+^ retention capacity (CRC) was defined as the amount of Ca^2+^ that mitochondria take up in small pulses before Ca^2+^ release.

### 2.9. Immunoblotting Analysis

HL-60 cells (5 × 10^6^ cells/mL) treated with MEL and/or navitoclax (ABT-737) were washed twice with ice-cold PBS, solubilized in lysis buffer containing 50 mM Tris-HCl (pH 7.4), 150 mM NaCl, 1% Triton X-100, 0.1% SDS, 1 mM EDTA, 1 mM Na_3_VO_4_, and 1 mM NaF and added proteinase/phosphatase inhibitors. The resulting samples were incubated on ice for 30 min and centrifuged at 13,000× *g* for 20 min at 4 °C. Protein concentration was measured by the Bradford method in supernatants that were dissolved in Laemmli Sample Buffer (Bio-Rad, Hercules, CA, USA), heated to 95 °C for 5 min. Prepared cell lysates were separated using 12.5% SDS-PAGE and transferred to a nitrocellulose membrane, blocked in Roti-block solution (Carl Roth GmbH + Co., Karlsruhe, Germany) at room temperature and after an hour incubated with the primary antibody at 4 °C overnight. The polyclonal Bcl_xL_, Bcl_w_, and Mcl antibodies were from Cell Signaling (Danvers, MA, USA) and the monoclonal C/EBP homologous protein (CHOP) and LC3A/B antibodies were from Cell Signaling (Danvers, MA, USA). The polyclonal antibodies to protein disulfide isomerase (PDI), binding immunoglobulin protein (BIP), ER oxidoreductin 1-Lα (ERO1-Lα), protein kinase R (PKR)-like endoplasmic reticulum kinase (PERK), and activating transcription factor 4 (ATF4) were from Cell Signaling (Danvers, MA, USA). The Glyceraldehyde 3-phosphate dehydrogenase (GAPDH) antibody (Cell Signaling, Danvers, MA, USA) was used as a loading control. The blot was detected by an ECL detection system (ChemiDoc Touch Imaging System, Bio-Rad, Hercules, CA, USA). Protein bands were quantified by densitometry (Image Lab program, Bio-Rad, Hercules, CA, USA).

## 3. Results

**Figure 1:**
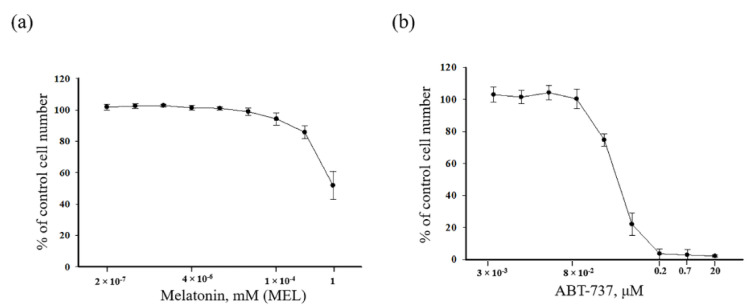
Concentration dependence of the cytotoxic effects of melatonin (MEL) and ABT-737. Cells were seeded in a 96-well plate at a density of 5 × 10^3^ cells per well and treated with indicated concentrations of (**a**) MEL and (**b**) ABT-737 for 24 h. The data are presented as means ± S.D. of ten separate experiments.

The cytotoxic effects of MEL (Figure 1a) and ABT-737 (Figure 1b) in HL-60 human leukemic cells were analyzed. Cells were treated with different concentrations of MEL (from 2 × 10^−7^ to 1 mM) and ABT-737 (from 3 × 10^−3^ to 20 μM) for 24 h. In the present work, we estimated two concentrations of the ABT-737 (Figure 1a) 0.7 and 0.2 μM. MEL had a significant effect on the viability of HL-60 cells at the concentration of 1 mM (as shown in Figure 1b).

**Figure 2:**
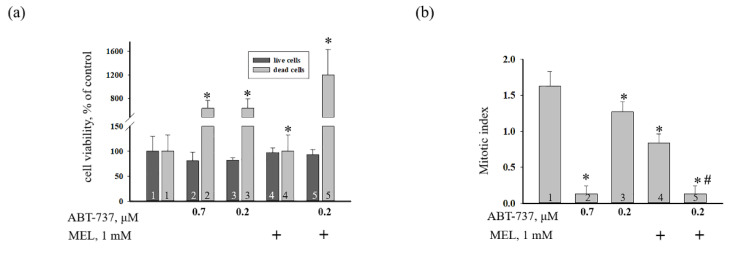
The effect of MEL and ABT-737 on the viability and proliferation of HL-60 cells. Cells were treated with 0.7 µM ABT-737 (column 2), and 0.2 µM ABT-737 (columns 3) and 1 mM MEL (column 4), and MEL in combination with 0.2 µM ABT-737 (column 5); untreated cells (control, column 1). (**a**) Cell viability in % relative to the control. (**b**) Mitotic index was calculated in the presence of 0.7 µM ABT-737 (column 2), and 0.2 µM ABT-737 (columns 3) and 1 mM MEL (column 4), and MEL in combination with 0.2 µM ABT-737 (column 5); untreated cells (control, column 1). “+” means the presence of MEL. The data are presented as the means ± S.D. of six separate experiments. * *p* < 0.05 significant difference in values in comparison with the control, ^#^ *p* < 0.05 significant difference in values compared to the value obtained after the addition of ABT-737 alone (0.2 µM, column 3).

The effect of MEL, ABT-737 (0.2 and 0.7 μM), and the combined effect of MEL and ABT-737 (0.2 μM) on cell death in HL-60 cells (Figure 2) were evaluated. Figure 2a demonstrates the viability of HL-60 cells under various conditions. We observed that the number of live cells was not changed in the presence of each compound, however, the number of death cells increased in the presence of ABT-737 (0.7 and 0.2 μM) by six times and MEL in combination with (ABT-737, 0.2 μM) by twelve times compared to the control. The mitotic index (MI) is considered a measure of the proliferation status of a cell population and is calculated as the ratio of the number of cells in mitosis to the total number of cells. We calculated the MI of HL-60 cells under our experimental conditions. Figure 2b shows the effect of compounds on the mitotic activity of cells. Figure 2b shows the effect of MEL and ABT-737 on the mitotic activity of cells. We found that MI decreased more than ten times in the presence of 0.7 μM ABT-737 (columns 2 vs. 1), by ~26% in the presence of 0.2 μM ABT-737 (columns 3 vs. 1), and by two times in the presence of MEL compared to control (without treatment) (columns 4 vs. 1). With the combined effect of the compounds, MI was reduced more than tenfold as compared with the control (without treatment) (columns 5 vs. 1) and by seven times when compared to experiments with MEL alone (columns 5 vs. 4). It should be noted that the value of the MI at the combined action of the MEL and 0.2 μM ABT-737 did not differ from that in the presence of 0.7 μM ABT-737 in HL-60 cells. The obtained results imply that MEL can enhance the cytotoxicity of ABT-737 at low concentrations in HL-60 human leukemic cells.

**Figure 3:**
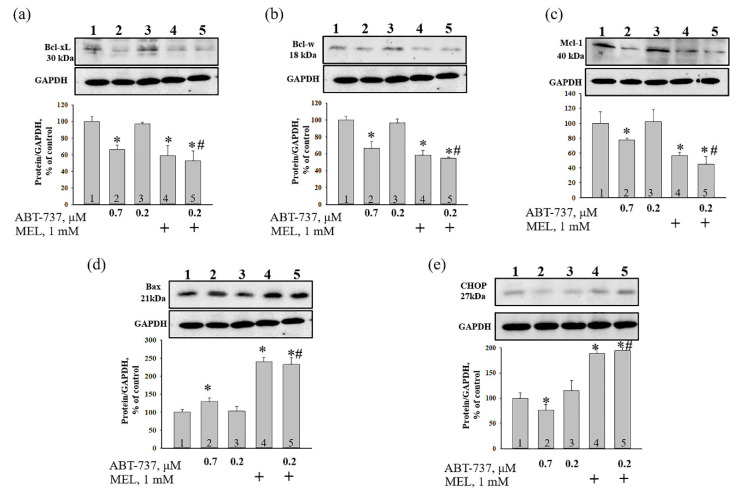
The effect of MEL and ABT-737 on the level of apoptosis-associated proteins—Bcl-xL (**a**) and Bcl-w (**b**), Mcl-1 (**c**), Bax (**d**) and Endoplasmic reticulum (ER) stress marker C/EBP homologous protein (CHOP) (**e**) in HL-60 cells. Cells were treated with 0.7 µM ABT-737 (column 2), and 0.2 µM ABT-737 (columns 3) and 1 mM MEL (column 4), and MEL in combination with 0.2 µM ABT-737 (column 5); untreated cells (control, column 1). The ration protein level to GAPDH was used as a loading control. “+” means the presence of MEL. The protein level in the cell lysate without any additives served as a control (100%). The data are presented as the means ± S.D. of three separate experiments. * *p* < 0.05 significant difference in the protein level compared with the corresponding control, ^#^ *p* < 0.05 significant difference in the protein level compared to ABT-737 alone (0.2 µM, column 3).

It is known that MEL is capable to modulate the induction of apoptosis in hyperthermia-exposed human leukemia (U937) [28,29] and HL-60 cells [30]. In addition, recently, we showed that MEL (1 mM) in combination with retinoic acid (10 nM) decreased Bcl-2 expression in HL-60 [25]. In the present work, we investigated the combined effect of ABT-737 and MEL on the change of expression of apoptosis-associated proteins such as Bcl_xL_, Bcl_w_, Mcl-1, and BAX and ER stress marker (CHOP) in HL-60 cells by western blot analysis (Figure 3). Figure 3a–e (upper parts) shows a Western blot stained with Bcl_xL_, Bcl_w_, Mcl-1, BAX, and CHOP. GAPDH was used as a loading control. Figure 3a–e (lower parts) show the immunostaining results obtained by computed densitometry and are presented as the ratio of proteins to GAPDH. We observed that expression of Bcl_xL_, Bcl_w_, and Mcl-1 decreased by ~30, 30, and 20%, respectively, in the presence of ABT-737 (0.7 μM) compared to control (Figure 3a–c, column 2 vs. 1), whereas ABT-737 (0.2 μM) did not change the level of each protein (Figure 3a–c, column 3 vs. 1). MEL diminished the expression of each protein by ~40% (Figure 3a–c, column 4 vs. 1), while the combined effect of MEL and ABT-737 (0.2 μM) decreased the expression of proteins by ~50, 45, and 55%, respectively (Figure 3a–c, column 5 vs. 1). MEL in combination with ABT-737 (0.2 μM) decreased the level of Bcl_xL_, Bcl_w_, and Mcl-1 by ~45, 45, and 60% relative to ABT-737 (0.2 μM) alone (Figure 3a–c, column 5 vs. 3). ABT-737 (0.7 μM) increased the expression of BAX by 30% compared to control (Figure 3d, column 2 vs. 1), whereas ABT-737 (0.2 μM) did not change the level of protein (Figure 3d, column 3 vs. 1). In HL-60 cells treated with MEL and MEL in combination with ABT-737 (0.2 μM) highly enhanced the level of BAX by 2.5 times relative to control (Figure 3d, column 4, 5 vs. 1). Moreover, MEL in combination with ABT-737 (0.2 μM) increased BAX expression 2.5 times compared to ABT-737 (0.2 μM) alone (Figure 3d, column 5 vs. 3). Since the C/EBP homologous protein (CHOP) is induced by ER stress and mediates apoptosis [31], we tested the change in the expression of this protein under our experimental conditions. According to our results, ABT-737 (0.2 μM) did not influence the expression of CHOP, while ABT-737 (0.7 μM) decreased the level of the protein by ~25% and MEL increased the level of protein by ~90% compared to control (Figure 3e, column 4 vs. 1). MEL in combination with ABT-737 (0.2 μM) enhanced CHOP expression by two times relative to control (3e, column 5 vs. 1) and by 60% in comparison with ABT-737 (0.2 μM) alone (Figure 3e, column 5 vs. 3).

The changing of autophagy pathways can influence the development of cancer cells and resistance to chemotherapy. It is known that MEL is able to modulate autophagy and display a protective effect under certain conditions by enhancing or inhibiting the autophagy process [32,33,34]. Lyamzaev et al. presented evidence that in HepG2 cells, dissipation of mitochondrial membrane potential by TPP^+^ ions and in cells of carcinoma, the stimulation of autophagy correlated with oxidative phosphorylation uncoupling [35].

**Figure 4:**
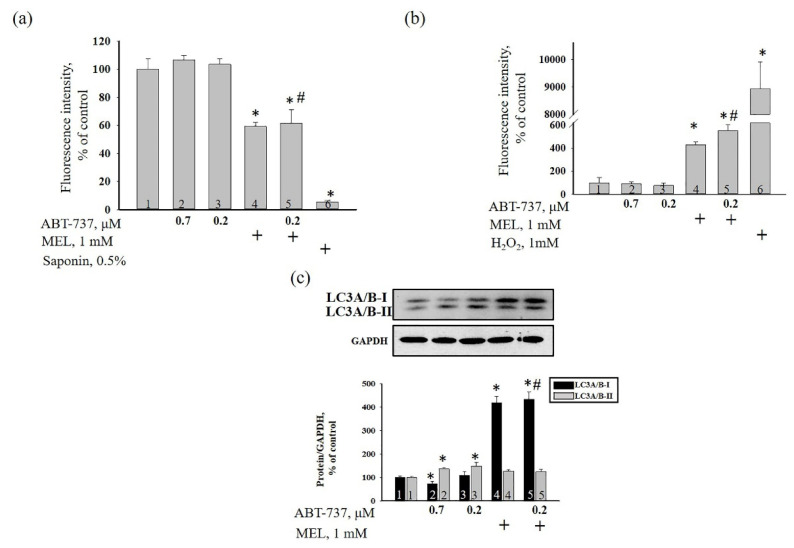
The effect of MEL and ABT-737 on the membrane potential (ΔΨ_m_) (**a**), ROS production (**b**) and in the level of autophagy marker LC3A/B (**c**) in HL-60 cells. Cells were treated with 0.7 µM ABT-737 (column 2), and 0.2 µM ABT-737 (columns 3) and 1 mM MEL (column 4), and MEL in combination with 0.2 µM ABT-737 (column 5); untreated cells (control, column 1). (**a**) the alteration of ΔΨ_m_ in our experimental conditions. Saponin (0.5%) was used as a positive control; (**b**) the alteration of ROS production in our experimental conditions. H_2_O_2_ (1 mM) was used as a positive control; (**c**) western blot of autophagy marker LC3A/B. The ratio of protein levels to GAPDH was used as a loading control. The protein level in the cell lysate without any additives served as a control (100%). “+” means the presence of MEL, Saponin and H_2_O_2_, respectively. The data are presented as the means ± S.D. of five separate experiments. * *p* < 0.05 significant difference in values compared with the corresponding control (untreated cells), ^#^ *p* < 0.05 significant difference in values relative to ABT-737 alone (0.2 µM, column 3).

Here, we analyzed the alterations in membrane potential (ΔΨ_m_), MEL-induced ROS production, and in the level of autophagy marker LC3A/B (I, II) in HL-60 cells in our experimental conditions (Figure 4). Figure 4a demonstrates the dissipation of the mitochondrial membrane potential. In MEL-treated cells, ΔΨ_m_ decreased by 40% compared to control (Figure 4a, column 4 vs. 1). MEL in combination with ABT-737 (0.2 μM) diminished ΔΨ_m_ by 40% relative to control or ABT-737 (0.2 μM) alone (Figure 4a, column 5 vs. 1; column 5 vs. 3). ABT-737 alone (0.7 and 0.2 μM) did not influence alteration of ΔΨ_m_. Figure 4b demonstrates the effect of MEL, ABT-737, and the combined action of MEL and ABT-737 on the oxidative activity in HL-60 cells. ABT-737 (0.7 and 0.2 µM) did not change DCFH-DA fluorescence intensity, reflecting ROS level in HL-60 cells. On the contrary, MEL strengthened DCFH-DA fluorescence intensity by four times compared to control (Figure 4b, column 4 vs. 1), therefore, increased the production of ROS in HL-60. We observed a similar effect with the combined action of MEL and ABT-737 (0.2 µM) in HL-60 cells relative to control, ROS level enhanced almost by five times (Figure 4b, column 5 vs. 1), by 28% compared to MEL alone (Figure 4b, column 5 vs. 4), and by seven times in comparison with ABT-737 (0.2 µM) alone (Figure 4b, column 5 vs. 3).

Figure 4c (upper part) showed a change in the level of LC3A/B (I, II) in HL-60 cells in our experimental conditions. Figure 4c (lower part) demonstrates the data on immunostaining obtained by computer-assisted densitometry and represents the ratios of proteins to GAPDH. We observed that the treatment of ABT-737 (0.7 μM) decreased the level of LC3A/B-I approximately by 20% compared to control (Figure 4b, black column 2 vs. 1) but LC3A/B-II expression increased by 30% (Figure 4b, grey column 2 vs. 1) in HL-60 cells. ABT-737 (0.2 μM) enhanced the expression of LC3A/B-II by 35% (Figure 4b, grey column 3 vs. 1) rather than LC3A/B-I level relative to control. Interesting to note that the treatment MEL and MEL in combination with ABT-737 (0.2 μM) increased the expression of LC3A/B-I by four times in HL-60 cells compared to control (Figure 4b, black column 4, 5 vs. 1). However, the level of LC3A/B-II did not change in these conditions.

**Figure 5:**
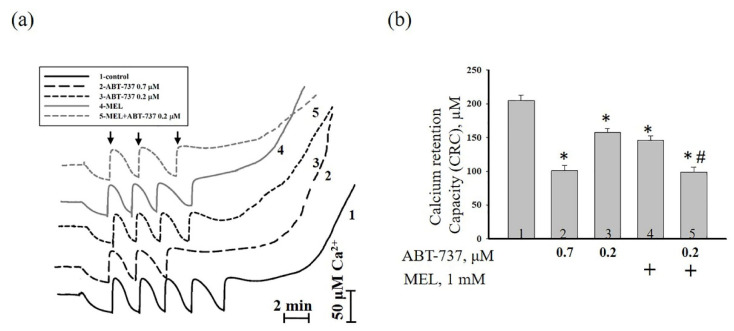
The effect of MEL and ABT-737 on the calcium retention capacity (CRC) in HL-60 cells. Cells were treated with 0.7 µM ABT-737 (column 2), and 0.2 µM ABT-737 (columns 3) and 1 mM MEL (column 4), and MEL in combination with 0.2 µM ABT-737 (column 5); untreated cells (control, column 1). (**a**) Ca^2+^ fluxes in HL-60 cells permeabilized with digitonin (0.007%). Arrows show the times at which CaCl_2_ (50 nmol of Ca^2+^ per mg of protein) was added; (**b**) Quantitative analysis of Ca^2+^ retention capacity corresponding to the threshold concentration of Ca^2+^. “+” means the presence of MEL The data are presented as the means ± S.D. of five separate experiments. * *p* < 0.05 significant difference relative to the control (untreated cells), ^#^ *p* < 0.05 significant difference compared to MEL alone (column 4).

Next, we checked calcium retention capacity (CRC) in our experimental conditions (Figure 5). Figure 5a demonstrates Ca^2+^ transport in the presence of different conditions. The fifth addition of Ca^2+^ led to Ca^2+^ release in control (curve 1). The treatment of ABT (0.7 μM, curve 2) and ABT (0.2 μM, curve 3) caused Ca^2+^ release after the third and fourth of Ca^2+^ addition. Ca^2+^ release was observed after the fourth addition of Ca^2+^ in the presence of MEL (curve 4), whereas the treatment of MEL in combination with ABT (0.2 μM) resulted in Ca^2+^ release after the third Ca^2+^ addition (curve 5). The value of CRC is shown in Figure 5b. In the presence of ABT (0.7 μM), CRC decreased by two times compared to control (Figure 5b, column 2 vs. 1), while CRC in the presence of ABT (0.2 μM) diminished by 25% (Figure 5b, column 3 vs. 1). The addition of MEL to HL-60 cells led to a decrease of CRC by 30% relative to control (Figure 5b, column 4 vs.1), whereas MEL in combination with ABT (0.2 μM) reduced this parameter by two times (Figure 5b, column 5 vs. 1). In the presence of MEL in combination with ABT (0.2 μM), CRC decreased by 32% compared to MEL alone (Figure 5b, column 5 vs. 4). MEL enhanced the effects of ABT (0.2 μM) in HL-60 cells.

**Figure 6:**
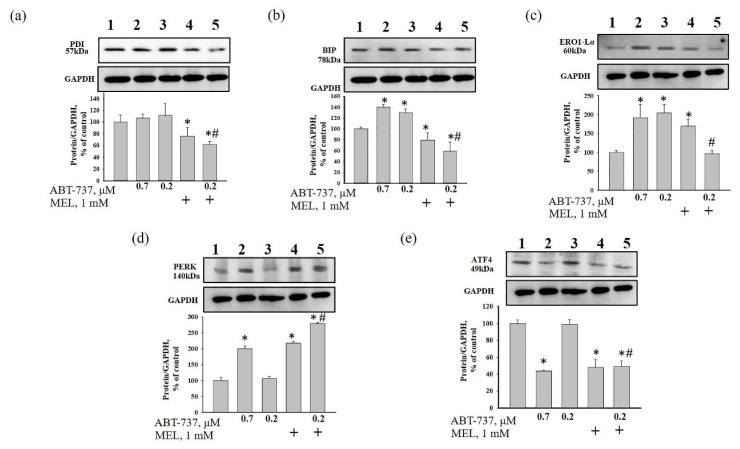
The combined effect of MEL and ABT-737 on the level of ER stress markers—Protein disulfide isomerase (PDI) (**a**), binding immunoglobulin protein (BIP) (**b**) ER oxidoreductin 1-Lα (ERO1-Lα) (**c**), protein kinase R (PKR)-like endoplasmic reticulum kinase (PERK) (**d**) and the activating transcription factor ATF4 (**e**) in HL-60 cells. Cells were treated with 0.7 µM ABT-737 (column 2), and 0.2 µM ABT-737 (columns 3) and 1 mM MEL (column 4), and MEL in combination with 0.2 µM ABT-737 (column 5); untreated cells (control, column 1). The ratio of protein levels to GAPDH was used as a loading control. The protein level in the cell lysate without any additives served as a control (100%). “+” means the presence of MEL The data are presented as the means ± S.D. of three separate experiments. * *p* < 0.05 significant difference in the protein level relative to the control (untreated cells), ^#^ *p* < 0.05 significant difference in the protein level compared to ABT-737 alone (0.2 µM, column 3).

There is a suggestion that MEL activates ER stress in some types of cancer cells [17,18,19]. Moreover, it was noticed that the increased level of ROS was able to cause ER stress in some conditions in colon cancer cells [36]. To assess the effect of MEL on ER stress in HL-60 cells in our experimental conditions, the expression of ER-stress associated proteins, such as PDI, BiP, ERO1-Lα, and PERK were examined (Figure 6a–d). In addition, we checked the alteration in the level of ATF4 in our experimental conditions (Figure 6e). MEL decreased the level of PDI by 25% relative to control (Figure 6a, column 4 vs. 1). MEL in combination with ABT-737 (0.2 µM) diminished the expression of PDI by 40% compared to control (Figure 6a, column 5 vs. 1), and by 50% relative to ABT-737 (0.2 µM) alone (Figure 6a, column 5 vs. 3). ABT-737 (0.7 µM) enhanced the level of BIP (by 40%) (Figure 6b, column 2 vs. 1) and ERO1-Lα (by 90%) (Figure 6c, column 2 vs.1), whereas ABT-737 (0.2 µM) had the same effect and increased the level of BIP by 30% (Figure 6b, column 3 vs. 1) and ERO1-Lα by two times compared to control (Figure 6c, column 3 vs. 1). However, MEL decreased BIP expression by 20% (Figure 6b, column 4 vs. 1) but increased the level of ERO1-Lα by 70% (Figure 6c, column 4 vs. 1) compared to control. MEL in combination with ABT-737 (0.2 µM) decreased BIP expression by 40% (Figure 6b, column 5 vs. 1) compared to control. The combined effect of MEL and ABT-737 (0.2 µM) diminished the levels of BIP by 70% (Figure 6b, column 5 vs. 3) and ERO1-Lα by two times (Figure 6c, column 5 vs. 3) relative to ABT-737 (0.2 µM) alone. The level of PERK was increased by two times in ABT-737 (0.7 µM)- and MEL-treated HL-60 cells (Figure 6d, column 2 vs. 1 and column 4 vs. 1), however MEL in combination with ABT-737 (0.2 µM) enhanced the level PERK by three times relative to control (Figure 6d, column 5 vs. 1) and ABT-737 (0.2 µM) alone (Figure 6d, column 5 vs. 3). The expression of ATF4 was decreased by ~55% and 50% in ABT-737 (0.7 µM)—and MEL-treated HL-60 cells, respectively, relative to control (Figure 6e, column 2 vs. 1 and 4 vs. 1). Combined effect of MEL and ABT-737 (0.2 µM) diminished the levels of ATF4 by 50% compared to control (Figure 6e, column 5 vs. 1) and ABT-737 (0.2 µM) (Figure 6e, column 5 vs. 3).

## 4. Discussion

Acute promyelocytic leukemia is an aggressive type of acute myeloid leukemia, which is a cancer of the blood and bone marrow. One of the most pressing problems in medicine is to found new approaches to the treatment of malignant tumors. Therefore, research in this area should be directed towards finding new drugs or combining drugs to prevent the development of malignant tumors. An ABT-737 inhibitor of Bcl_xL_, Bcl-2 and Bcl_w_ has been described. It is known that ABT-737 binds to the hydrophobic moiety in these proteins and prevents them from sequestering pro-apoptotic proteins such as BAD and BIM [23]. Therefore, it has been suggested that ABT-737 is capable of causing cell death after treatment with agents that induce pro-apoptotic signals [23]. Since ABT-737 exhibits significant monotherapeutic activity against myeloma and leukemia [23,24], studies of this drug are of great interest. The indolamine secreted by the pineal gland known as melatonin has shown potential for use as an adjuvant in cancer treatment by enhancing the therapeutic effects and reducing the side effects of chemotherapy or radiation [37]. It is known that in normal cells MEL is an antioxidant and exhibits anti-apoptotic properties [38,39,40], while in tumor cells, on the contrary, MEL exhibits pro-apoptotic properties and is able to inhibit the growth and development of cancer tumors [41]. Recently, we showed that MEL in combination with retinoic acid or cytarabine decreased viability and mitotic index and increased cytotoxicity toward HL-60 cells and suppressed the expression of anti-apoptotic Bcl-2 protein [25,26]. In the present study, we investigated the combined effect of MEL and ABT-737 with a reduced concentration on the viability of HL-60 cells, the alterations in the level of anti- and pro-apoptotic proteins such as Bcl_xL_, Bcl_w_, Mcl-1, and BAX. We observed that MEL in combination with ABT-737 (0.2 µM) decreased mitotic index. The changes in the mitotic index in HL-60 cells under the combined effect of ABT-737 (0.2 µM) with MEL (1 mM) are similar to those that were observed by the influence of ABT-737 (0.7 µM) (Figure 2b). ABT-737 associates with high affinity to Bcl-2, Bcl_xL_, Bcl_w_ and inhibits them, and with low affinity to Mcl-1 and A1, respectively. Apoptosis of tumor cells under the influence of ABT-737 can be induced if the cell contains a sufficient amount of BAX and Bak [42,43]. Here, the expression of Bcl_xL_, Bcl_w_, and Mcl-1 decreased in the presence of MEL in combination with ABT-737 (0.2 µM) relative to control, whereas the expression of BAX increased. This effect was similar to the effect of ABT-737 (0.7 μM) alone on HL-60 cells (Figure 3). CHOP is induced by ER stress mediates apoptosis [31]. It is known that medium ER stress is protective, however, when ER stress becomes irreversible and normal function cannot be restored, an apoptotic signal is initiated; thus, cells eventually induce programmed cell death. ER stress-induced apoptosis involves the expression/activation of ER stress-associated pro-apoptotic molecules, including CHOP. This protein can activate apoptotic proteins downstream of the ER stress, including Bcl-2 proteins (Bcl_xL_, Bcl_w_, and Mcl-1) and BAX [44]. In our study, after treatment with MEL and MEL alone in combination with ABT-737 (0.2 μM), CHOP expression increased, which suggests the activation of ER stress, which triggers the apoptosis signaling cascade. The mitochondrial transition pore (mPTP) refers to an alteration in the permeability of the inner mitochondrial membrane and is considered the initial stage of apoptosis [45]. The opening of mPTP is accompanied by the drop of mitochondrial membrane potential, depolarization of the mitochondrial membrane, and Ca^2+^ release from the mitochondrial matrix. We observed that the treatment with MEL in combination with ABT-737 (0.2 μM) resulted in an acceleration of mPTP opening and, therefore, a decrease of Ca^2+^ retention capacity in HL-60 cells (Figure 5).

Apoptosis and autophagy are considered to be programmed cell death processes that act independently of each other in many biological actions [18,33,46,47,48]. MEL is thought to facilitate and control autophagy in cancer cells in a variety of ways, depending on their ability to activate apoptosis [49]. Moreover, Dauchy and coauthors showed that MEL caused autophagy in breast tumor xenografts, leading to tumor regression [50]. In another study, it was exhibited that, MEL in combination with sorafenib enhanced mitophagy, due to its action in increasing mitochondrial depolarization and ROS production in hepatocellular carcinoma cells [51]. In our work, MEL and MEL in combination with ABT-737 (0.2 μM) caused depolarization of the mitochondrial membrane, increasing the drop in membrane potential, which could lead to an increase in ROS production with a subsequent enhancement in autophagy (Figure 4).

It is known that autophagy may result from ER stress [20]. Since the endoplasmic reticulum is involved in many cellular functions, namely calcium homeostasis, protein or phospholipid synthesis [52] and also regulates the activation of various transcriptional cascades, intracellular transport, mitochondrial biogenesis, autophagy and apoptosis [53,54,55], we checked the change in the expression of proteins in our experimental conditions. Unfolded protein response is mediated by three membrane-bound ER proteins (PERK, ATF6 and IRE1), which are usually inactivated by the BiP chaperone. The interaction of BiP with unfolded luminal proteins, the accumulation of which causes ER stress, activates PERK, ATF6, and IRE1 and completes the transcription of genes associated with autophagy through the activation of XBP1, CHOP, and ATF4 [56]. In our experimental conditions, MEL and MEL in combination with ABT-737 (0.2 μM) increased the expression of PERK, resulting in a decrease in the level of BiP followed by an increase in the level of CHOP but not ATF4.

CHOP is associated with apoptosis through modulation of pro-apoptotic and anti-apoptotic proteins and participated in the release of Ca^2+^ from the endoplasmic reticulum by ERO1 [57]. The level of CHOP increased in the presence of MEL and MEL in combination with ABT-737 (0.2 μM) (Figure 3e) that can lead to a decrease of the expression of ERO1 (Figure 6d) and Ca^2+^ capacity in HL-60 cells (Figure 5).

It should be noted that BiP and PDI are required to maintain Ca^2+^ homeostasis [58]. Moreover, PDI has a disulfide isomerase activity and an oxidase activity to form disulfide bonds, involving protein folding and isomerization, and promoting increased ROS in cells [59]. The oxidized form of PDI is restored to maintain its oxidase capacity with the aid of ERO1, which uses oxygen as the final electron acceptor [57]. In our study, MEL and MEL in combination with ABT-737 (0.2 μM) decreased the expression ERO1, which could affect the decrease in the level of PDI. A decrease in Ca^2+^ capacity and drop of membrane potential correlated with enhancement of ROS production and a decrease of PDI expression in the presence of MEL in combination with ABT-737 (0.2 μM) in HL-60 cells.

## 5. Conclusions

Melatonin has a beneficial effect in several pathophysiological conditions, including various types of cancer. It should be noted that in cancer cells, melatonin exhibits a pro-apoptotic effect, while in normal cells it is anti-apoptotic. In the present study, the combined effect of melatonin and ABT-737 (0.2 μM) on the alteration of pro- and anti-apoptotic proteins, the ER stress, Ca^2+^ retention capacity, the drop of mitochondrial membrane potential, and autophagy was examined in HL-60 cells. We observed that MEL in combination with ABT-737 (0.2 μM) decreased Ca^2+^ capacity, dropped membrane potential, increased ROS production, suppressed the expression of anti-apoptotic proteins such as Bcl_xL_, Bcl_w_, and Mcl-1, and enhanced the expression pro-apoptotic of BAX. These changes suggest the initiation of the apoptosis signaling cascade. Moreover, MEL in combination with ABT-737 (0.2 μM) caused depolarization of the mitochondrial membrane, increasing the drop of membrane potential, which could lead to an increase in ROS production with a subsequent enhancement in autophagy. Changes in the expression of proteins implicated in stress by the combined action of melatonin and ABT-737 suggest that melatonin in combination with anti-tumor compounds has potential usefulness in the treatment of cancer, where it is able to modulate ER stress, autophagy and apoptosis.
